# Genome-wide Association Mapping of Cold Tolerance Genes at the Seedling Stage in Rice

**DOI:** 10.1186/s12284-016-0133-2

**Published:** 2016-11-15

**Authors:** Dan Wang, Jinling Liu, Chengang Li, Houxiang Kang, Yue Wang, Xinqiu Tan, Minghao Liu, Yufei Deng, Zhilong Wang, Yong Liu, Deyong Zhang, Yinghui Xiao, Guo-Liang Wang

**Affiliations:** 1 Southern Regional Collaborative Innovation Center for Grain and Oil Crops in China and College of Agronomy, Hunan Agricultural University, Changsha, Hunan 410128 China; 2 Institute of Plant Protection, Hunan Academy of Agricultural Sciences, Changsha, Hunan 410128 China; 3 State Key Laboratory for Biology of Plant Diseases and Insect Pests, Institute of Plant Protection, Chinese Academy of Agricultural Sciences, Beijing, 100193 China; 4 Department of Plant Pathology, Ohio State University, Columbus, OH 43210 USA

**Keywords:** Oryza sativa, Cold tolerance, Quantitative trait locus (QTL), GWAS

## Abstract

**Background:**

Rice is a temperature-sensitive crop and its production is severely affected by low temperature in temperate and sub-tropical regions. To understand the genetic basis of cold tolerance in rice, we evaluated the cold tolerance at the seedling stage (CTS) of 295 rice cultivars in the rice diversity panel 1 (RDP1), these cultivars were collected from 82 countries.

**Results:**

The evaluations revealed that both temperate and tropical japonica rice cultivars are more tolerant to cold stress than indica and AUS cultivars. Using the cold tolerance phenotypes and 44 K SNP chip dataset of RDP1, we performed genome-wide association mapping of quantitative trait loci (QTLs) for CTS. The analysis identified 67 QTLs for CTS that are located on 11 chromosomes. Fifty-six of these QTLs are located in regions without known cold tolerance-related QTLs.

**Conclusion:**

Our study has provided new information on the genetic architecture of rice cold tolerance and has also identified highly cold tolerant cultivars and CTS-associated SNP markers that will be useful rice improvement.

**Electronic supplementary material:**

The online version of this article (doi:10.1186/s12284-016-0133-2) contains supplementary material, which is available to authorized users.

## Background

Temperature adaptability is a critical factor for rice domestication and production. The differentiation of two major domesticated rice subspecies (indica and japonica) is associated with temperature. Japonica is tolerant to low temperature and is mainly planted in the temperate, sub-temperate and high altitude areas of the subtropics; indica, in contrast, is sensitive to low temperature and is mainly grown in tropical and sub-tropical areas (Zhao et al. 2013). Cold stress is often a major limiting factor for stable rice production in temperate areas and sub-tropic areas with high altitude. Cold stress restricts the growth and development of rice at all the growth stages and results in low germination and seedling vigor, delayed seedling growth with leaf wilting and browning, prolonged duration of cultivation, pollen sterility, poor grain filling, and reduced yields (Suh et al. 2010; Pan et al. 2015).

Rice cold tolerance is genetically controlled by multiple quantitative trait loci (QTLs). Traditional QTL mapping using bi-parental or multiple cross populations identified more than 250 QTLs on all 12 chromosomes for rice cold tolerance at different growth and development stages (Yang et al. 2015; Xiao et al. 2015; Mao et al. 2015; Zhu et al. 2015). Among these QTLs, several genes have been fine mapped, including *Ctb1* (Saito et al. 2004), *qCT8* (Kuroki et al. 2007), *qCTB7* (Zhou et al. 2010), *qCTB3* (Shirasawa et al. 2012), and *qCT-3-2* (Zhu et al. 2015) for cold tolerance at the booting stage, *qCTS12* (Andaya and Tai, 2006), *qCTS4* (Andaya and Tai, 2007), *qCtss11* (Koseki et al. 2010), *qSCT1* and *qSCT11* (Kim et al. 2014), *qLOP2* and *qPSR2-1* (Xiao et al. 2015) for CTS, *qLTG3-1* for germination cold tolerance (Fujino et al. 2008), and *qRC10-2* for root cold tolerance (Xiao et al. 2014). Two QTLs for rice cold tolerance, *Ctb1* and *COLD1*, have been cloned and functionally characterized (Saito et al. 2010; Ma et al. 2015). *Ctb1* is the first cloned QTL for rice cold tolerance and confers enhanced cold tolerance at the booting stage. *Ctb1* encodes a F-box protein and physically associates with Skp1, a subunit of the E3 ubiquitin ligase, suggesting the potential involvement of the ubiquitin–proteasome pathway in rice cold resistance (Saito et al. 2010). The newly identified *COLD1* gene confers cold tolerance in japonica rice at the seedling stage. Molecular characterization revealed that *COLD1* functions as a GTPase-accelerating factor and regulates G-protein signaling by sensing cold in order to trigger Ca^2+^ signaling for cold tolerance (Ma et al. 2015).

Genome-wide association analysis (GWAS) was applied for QTL mapping using large germplasm collections (Huang et al. 2010; Zhao et al. 2011). Many QTLs for multiple traits were identified, such as traits associated with agronomic characteristics (Huang et al. 2010; Zhao et al. 2011; Yang et al. 2014), and with responses to abiotic stresses (Famoso et al. 2011; Pan et al. 2015; Lv et al. 2016), and to biotic stresses (Jia et al. 2012; Wang et al. 2014; Kang et al. 2016; Wang et al. 2015). Using GWAS, Pan et al. (2015) recently mapped 51 QTLs for cold tolerance at the germination and booting stages with 174 Chinese rice accessions that were genotyped with 273 SSR markers. Fujino et al. (2015) also identified 17 QTLs responsible for rice low temperature germinability in 63 Japanese varieties genotyped with 115 SSR markers and two other markers. In addition, Lv et al. (2016) used 527 rice cultivars to identify 132 QTLs for both rice natural chilling and cold shock stresses.

In this study, we used GWAS to map QTLs associated with rice cold tolerance at the seedling stage (CTS). The GWAS involved 295 rice cultivars in the publically available rice diversity panel 1 (RDP1), these cultivars were collected from 82 countries and genotyped with a 44 K SNP chip (Zhao et al. 2011). The cold tolerance evaluations showed that both temperate and tropical japonica rice cultivars are more tolerant of cold stress than indica and AUS rice cultivars. A total of 67 QTLs associated with CTS were mapped on 11 chromosomes in the rice genome. These QTLs explained from 3.8 to 8.2% of the CTS. The mapped QTLs with corresponding linked SNP markers will be useful for the improvement of rice cold tolerance.

## Results

### Phenotypic Variation among RDP1 Seedlings in Response to Cold Treatment

**Fig. 1:**
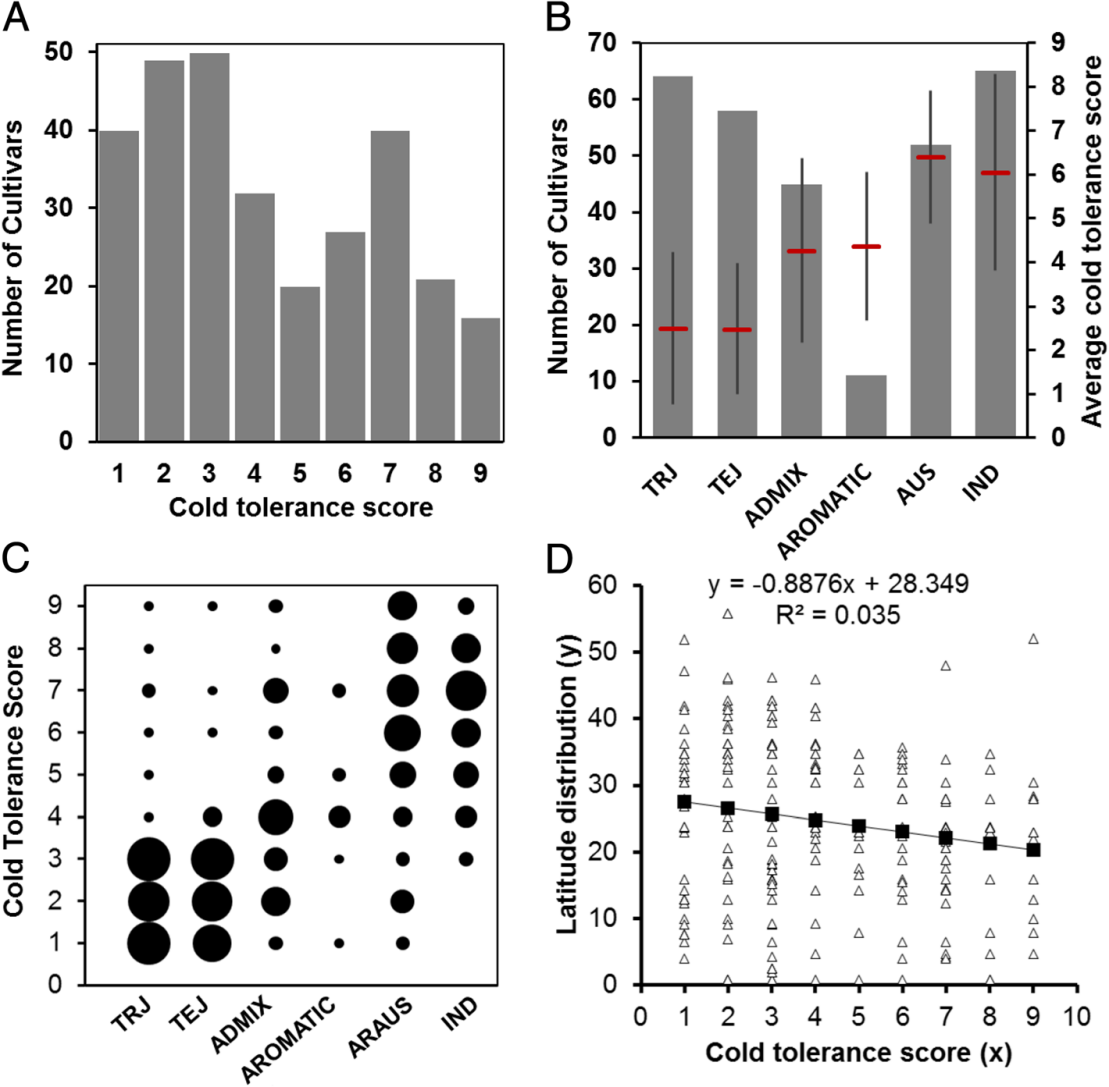
Phenotypic structure of the rice diversity panel1 (RDP1) in response to cold treatment. **a** The phenotype distribution of 295 rice accessions. The X-axis is cold tolerance scores (1–9); Y-axis represents the numbers of cultivars of each score. **b** The rice accession numbers (*left Y-axis*) of each subspecies (X-axis) used in the study and the average cold tolerance scores (*right Y-axis*) with error bars (STDE) for each subspecies. **c** The cold tolerance score structure (Y-axis) of rice accessions in each subspecies (X-axis). The circle size represents the number of rice cultivars for the corresponding cold tolerance scores (Y-axis) in different subspecies (X-axis). **d** Correlation analysis between rice cold tolerance scores and its latitude distribution by linear regression analysis. X-axis is the cold tolerance score of tested rice cultivars. Y-axis is latitude of the cultivars. Light triangle is the latitude value of a cultivar in Y-axis with the corresponding cold tolerance score in X-axis. Black square linked with the line is the predicted latitude value. The predicted formula and R^2^ are shown on the top

To assess the phenotypic variation in the cold tolerance of RDP1 cultivars, we evaluated 295 cultivars at the 3-leaf seedling stage. The cold tolerance scores of these cultivars are listed in Additional file 1: Table S1. About 60% of the cultivars were tolerant (scores 1–4) and about 40% were sensitive (scores 5–9) (Fig. 1a; Additional file 2: Table S2). The RDP1 collection consists of 6 subpopulations including 64 tropical japonica (TRJ), 58 temperate japonica (TEJ), 45 ADMIX, 11 AROMATIC, 52 AUS and 65 indica (IND) cultivars (Fig. 1b). The TEJ and TRJ groups had the highest CTS with average scores of 2.5 and 2.5, respectively. The AUS and IND groups had the lowest CTS with average scores of 6.4 and 6.1, respectively. The CTS was intermediate for the ADMIX and AROMATIC groups (Fig. 1b). High cold tolerance (score ≤ 3) was exhibited by 57 cultivars in the TRJ group and by 51 in the TEJ group, whereas low cold tolerance (score > 4.0) was exhibited by 51 cultivars in the IND group and by 45 in the AUS group (Fig. 1c). These results suggested that both temperate and tropical japonica rice cultivars are tolerant of cold stress and that the indica and AUS cultivars are sensitive to cold stress.

To further investigate the relationship between the cold tolerance level of world rice accessions and their geographical distribution, we performed a correlation coefficient analysis between the latitude of the cultivars and their cold tolerance scores. The analysis showed that a negative correlation existed between cultivars’ latitude and the level of their cold tolerance with a correlation coefficient at −0.188. In addition, the linear regression analysis confirmed the negative correlation (Fig. 1d).

### Mapping of QTLs for CTS by GWAS

**Fig. 2:**
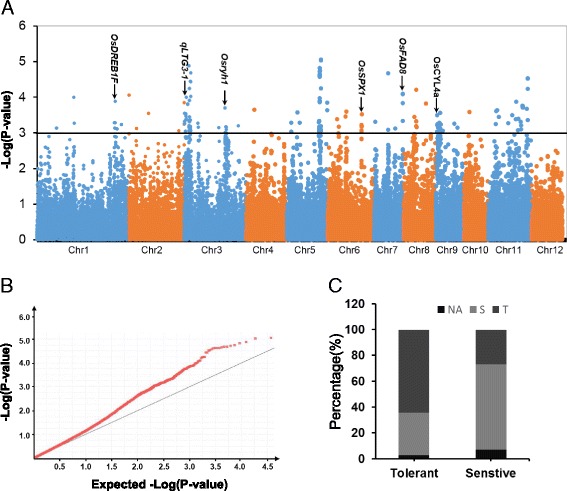
GWAS mapping of rice cold tolerance at seedling stage (CTS). **a** The Manhattan plots of CTS on rice chromosomes analyzed with the data of the RPD1 rice accessions. X-axis is the genomic coordinates. Y-axis is the LOD score for each SNP. **b** The corresponding Q-Q plots for GWAS mapping. **c** The average allele frequency of the 181 significant SNPs in the 40 highly tolerant and the 19 extremely sensitive rice cultivars. X-axis is the tolerant or sensitive rice group and Y-axis means the average SNP frequency of the corresponding allele genotype. T is tolerant associated SNP genotype, S represents sensitive SNP genotype, NA means unknown genotype

We performed GWAS using the CTS data and the 44 K SNP dataset published by Zhao et al. (2011) following the criteria of one associated locus between any two significant SNPs within 200 kb interval by Lv et al. (2016). The analysis revealed that 67 QTLs within 181 SNPs on 11 chromosomes were significantly associated with CTS with a well-fitted Quantile-Quantile (Q-Q) plots (Fig. 2a and b; Table 1 and Additional file 3: Table S3). The phenotypic contribution of each QTLs varied from 3.82 to 8.20% (Table 1). A comparison of previous results of cold tolerance gene mapping or functional characterization indicated that 11 associated loci were co-localized with the known, mapped QTLs or characterized genes (Additional file 3: Table S3). Among the co-localized loci, six known functional genes were *OsDREB1F* on chromosome 1 (Wang et al. 2008), *qLTG3-1* on chromosome 3 (Fujino et al. 2008), *OsRAN2* (Chen et al. 2011), *OsSPX1* on chromosome 6 (Zhao et al. 2009), *OsFAD8* on chromosome 7 (Nair et al. 2009), and *OsCYL4a* on chromosome 9 (Qin et al. 2015) (Fig. 2a; Additional file 3: Table S3). The remaining 56 QTLs were considered to be novel.

**Table 1:** The mapped QTLs for rice cold tolerance at seedling stage

QTLs	Chromosome	linked SNP loci^a^	linked tolerant SNP marker	LOD	Phenotype contribution	Candidate genes
*qCTS1-1*	1	7392085	CC	3.13	5.15	NA
*qCTS1-2*	1	19180885	TT	3.99	5.14	NA
*qCTS1-3*	1	20261058	AA	3.27	4.16	NA
*qCTS1-4*	1	41385263	TT	3.28	4.43	NA
*qCTS1-5*	1	41859177	AA	3.88	4.92	NA
*qCTS1-6*	1	42438945	CC	3.23	4.11	OsDREB1F, LOC_Os01g73770
*qCTS2-1*	2	74241	GG	4.05	5.45	NA
*qCTS2-2*	2	4797319	AA	3.12	3.85	NA
*qCTS2-3*	2	15096404	AA	3.54	4.44	NA
*qCTS2-4*	2	35929494	TT	3.84	5.07	NA
*qCTS3-1*	3	604512	CC	3.54	4.47	qLTG3-1, LOC_Os03g01320 Chr03:219977-221070
*qCTS3-2*	3	995211	TT	3.52	4.62	NA
*qCTS3-3*	3	1243161	AA	3.99	5.12	NA
*qCTS3-4*	3	2110407	TT	3.81	4.83	NA
*qCTS3-5*	3	2680925	CC	4.88	8.20	NA
*qCTS3-6*	3	3032938	TT	3.44	4.43	NA
*qCTS3-7*	3	3211019	CC	3.91	5.60	NA
*qCTS3-8*	3	3668304	CC	4.68	6.14	NA
*qCTS3-9*	3	3763778	GG	4.43	5.74	*Osryh1*, LOC_ Os03g019140
*qCTS3-10*	3	8192574	AA	3.26	4.04	NA
*qCTS3-11*	3	22540833	TT	3.14	3.90	NA
*qCTS3-12*	3	27608937	AA	3.69	5.96	NA
*qCTS3-13*	3	31924530	AA	3.19	3.97	NA
*qCTS4-1*	4	5850082	TT	3.65	4.59	NA
*qCTS5-1*	5	1970193	GG	3.03	4.44	NA
*qCTS5-2*	5	6171822	CC	3.58	4.51	NA
*qCTS5-3*	5	8040996	AA	3.29	4.51	NA
*qCTS5-4*	5	24616513	GG	3.33	4.18	NA
*qCTS5-5*	5	25098060	TT	4.56	6.25	NA
*qCTS5-6*	5	25154581	AA	4.82	6.39	NA
*qCTS5-7*	5	25720662	AA	3.1	4.00	NA
*qCTS5-8*	5	26092015	TT	5.05	6.77	NA
*qCTS5-9*	5	29432186	TT	3.84	4.97	NA
*qCTS6-1*	6	4733861	TT	3.39	4.25	NA
*qCTS6-2*	6	6228690	AA	3.18	4.02	NA
*qCTS6-3*	6	10452388	CC	3.46	5.81	NA
*qCTS6-4*	6	10964887	GG	3.6	4.55	NA
*qCTS6-5*	6	23876111	TT	3.52	4.42	OsSPX1, LOC_Os06g40120
*qCTS7-1*	7	914685	CC	3.3	4.13	NA
*qCTS7-2*	7	17875297	GG	3.09	3.82	NA
*qCTS7-3*	7	18202939	CC	4.67	7.06	NA
*qCTS7-4*	7	23783174	CC	3.12	3.92	NA
*qCTS7-5*	7	29220680	AA	4.08	5.34	OsFAD8 LOC_Os07g49310
*qCTS8-1*	8	8883364	AA	3.44	4.34	NA
*qCTS8-2*	8	10142772	CC	4.21	5.48	NA
*qCTS8-3*	8	10506862	GG	3.16	3.91	NA
*qCTS8-4*	8	20385371	AA	3.82	4.97	NA
*qCTS9-1*	9	1141448	CC	3.49	4.40	OsCYL4a, LOC_Os09g02270
*qCTS9-2*	9	1706118	CC	3.25	4.33	NA
*qCTS9-3*	9	3375476	CC	3.29	4.97	NA
*qCTS9-4*	9	3869544	GG	3.11	4.57	NA
*qCTS9-5*	9	4289803	GG	3.56	4.48	NA
*qCTS9-6*	9	4767843	TT	3.04	4.20	NA
*qCTS9-7*	9	6403821	AA	3.06	4.65	NA
*qCTS9-8*	9	16181745	CC	3.26	4.04	NA
*qCTS9-10*	9	17697057	GG	3.15	3.89	NA
*qCTS10-1*	10	5690202	CC	3.59	4.63	NA
*qCTS11-1*	11	5902436	AA	3.25	4.15	NA
*qCTS11-2*	11	6478793	GG	3.6	5.02	NA
*qCTS11-3*	11	7249274	GG	3.64	4.61	NA
*qCTS11-4*	11	9163345	GG	3.45	4.33	NA
*qCTS11-5*	11	17519565	AA	3.27	4.68	NA
*qCTS11-6*	11	19897968	AA	3.49	4.95	NA
*qCTS11-7*	11	21779484	AA	3.87	6.75	NA
*qCTS11-8*	11	22115297	GG	3.21	5.11	NA
*qCTS11-9*	11	24958836	CC	3.67	4.68	NA
*qCTS11-10*	11	25432012	CC	4.52	6.48	NA

Note: ^a^only the linked SNP loci with highest LOD scores are shown. NA means not available

Next, we evaluated the allele frequency of the 181 significant SNPs in the 40 highly tolerant rice cultivars (score = 1) and the 19 extremely sensitive cultivars (score = 9) (Additional file 4: Table S4). The analysis showed that the highly tolerant rice accessions contained an average of 64.3% of the tolerant alleles and 33.1% of the sensitive types (Fig. 2c). In contrast, the highly sensitive cultivars only contained 26.9% of the tolerant alleles and 66.3% of the sensitive types (Fig. 2c). These results demonstrate that the identified 181 significant SNPs have a large contribution to the cold tolerance of the tested RPD1 cultivars.

### Identification of Candidate Genes Responsible for CTS

**Fig. 3:**
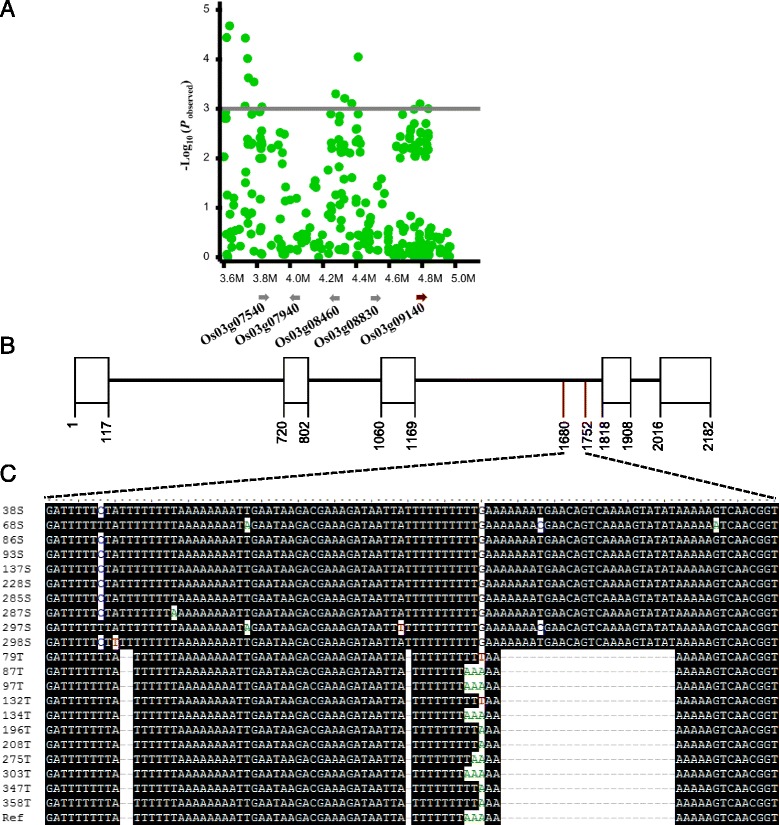
Validation of the rice cold tolerance QTL *qCTS3-9*. **a** Candidate gene prediction of *qCTS3-9*. **b** The gene encoding structure of candidate gene LOC_Os03g019140. **c** Sequence alignment of LOC_Os03g019140 gene in the indel region between cold tolerant and sensitive accessions. T means tolerant, S is sensitive, Ref is the reference sequence of Nipponbare genome with an tolerant phenotype

To identify candidate genes that are localized in the CTS QTL regions, we analyzed a 500 kb genomic region by comparing the QTL regions with the Nipponbare reference genome. Interestingly, we found that the candidate gene LOC_Os03g09140 on chromosome 3, designate *qCTS3-9* (Fig. 2a and Additional file 3: Table S3), is *Osryh1*, which is linked to the NP:rs19079958 (*p* = 9.61 × 10^−5^) (Fig. 3a). *Osryh1* encodes a GTP-binding protein and is a homolog of the yeast *ryh1* gene, which is responsible for temperature sensing in yeast (Bednarek et al. 1994). However, the function of *Osryh1* in cold tolerance is not clear.

**Fig. 4:**
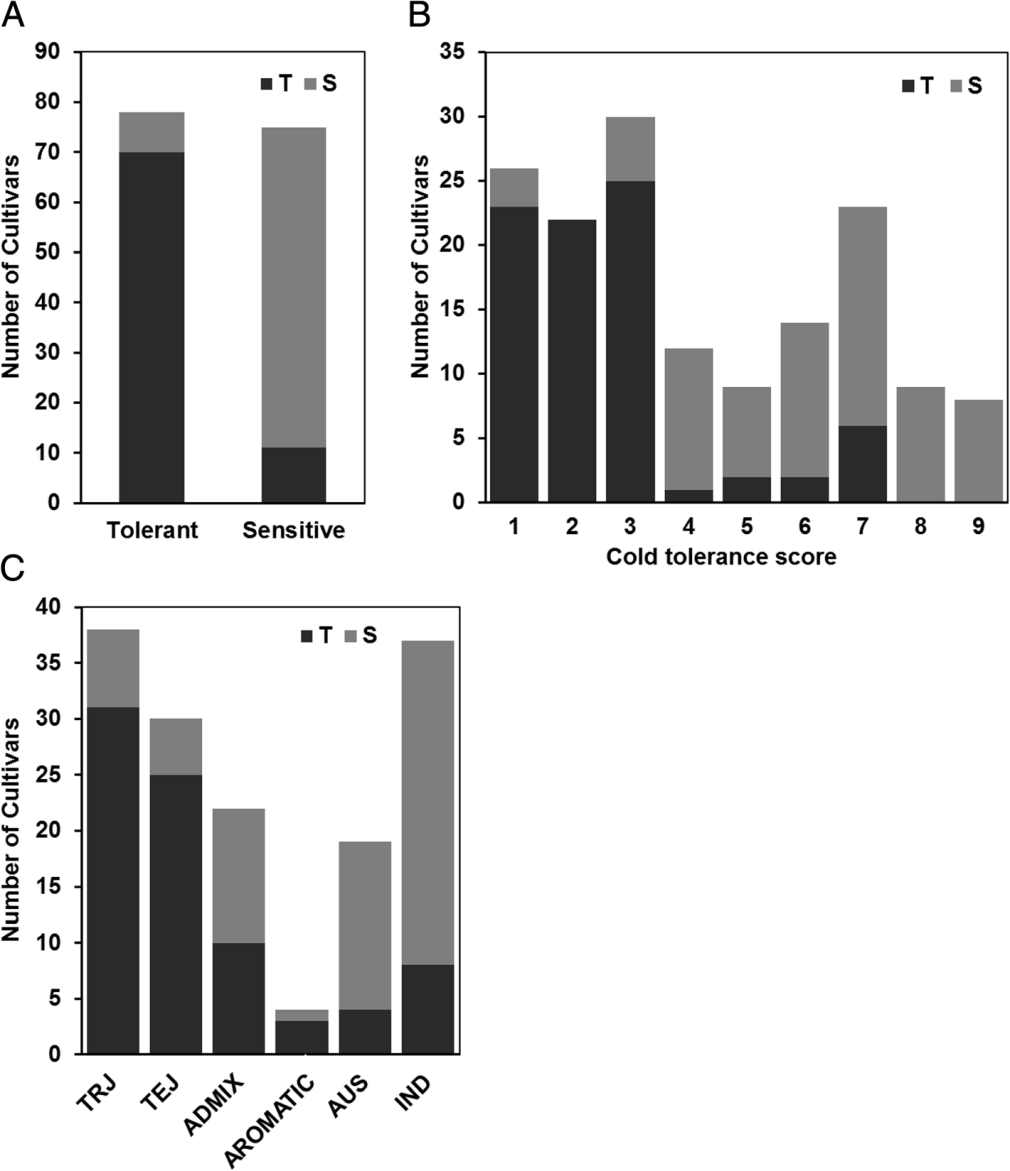
Linkage confirmation of *qCTS3-9* in the rice collection using a newly developed indel marker. **a** The distribution of tolerant (T) genotype (*black box*) without the 27 bp sequence and sensitive (S) genotype (*gray box*) with the 27 bp sequence in 153 rice accessions. **b** The distribution of T and S types in each cold tolerance score. C. The distribution of T and S types in each subspecies

To confirm the association between *Osryh1* and the CTS phenotype, we sequenced *Osryh1* in 10 cold-tolerant and 10 cold-sensitive cultivars. The sequence analysis showed that the tolerant cultivars contained three deletions in the intron 3 with a total length of 27 bp comparing with the corresponding sequence of sensitive cultivars (Fig. 3b, c). Furthermore, we designed an indel marker using the 27-bp deletion sequence to genotype 153 RDP1 accessions (78 tolerant and 75 sensitive cultivars). The results showed that 89.74% (70 out of 78) tolerant cultivars (score less than or equal to 3) had tolerant alleles, and 85.3% (64 out of 75) sensitive cultivars (score more than or equal to 4) had sensitive alleles (Fig. 4a, b). In addition, 83.3% (25 out of 30) tolerant temperate japonica cultivars and 81.6% (31 out of 38) tolerant tropical had the deletions, and 78.4% (29 out of 37) sensitive IND cultivars and 78.9% (15 out of 19) sensitive AUS cultivars did not contain the deletions (Fig. 4c).

## Discussion

In this study, we evaluated the genetic structure of cold tolerance in 295 RDP1 rice cultivars at the seedling stage. Consistent with a previous study (Pan et al. 2015), we found that both temperate and tropical japonica cultivars are more tolerant to cold stress than the IND and AUS cultivars (Fig. 1 and Additional file 1: Table S1). Interestingly, we found that the tropical rice cultivars, also known as javanica, show a high level of tolerance to cold stress. Although a study indicated that tropical japonica cultivars are tolerant to cold stress (Suh et al. 2010), a comprehensive evaluation of tropical japonica rice cultivars have not been reported. Our results revealed that 89% of the 64 TRJ rice cultivars from 40 countries are highly tolerant to cold stress (Additional file 2: Table S2), suggesting that tropical rice is also an important germplasm for rice cold tolerance improvement.

By evaluating the natural chilling and cold shock stresses with a panel of rice accessions from China, Lv et al. (2016) recently found that the latitudinal distribution of rice is correlated with natural cold adaptability. In this study, we also observed a negative correlation between latitudinal distribution and rice cold tolerance level among the RDP1 accessions (Fig. 1d). This result suggests that high latitude could be one of the selected environmental factors for domestication of cold tolerant rice cultivars. Additonaly, 40 RDP1 rice cultivars showed extremely high tolerance to cold stress (Additional file 2: Table S2), and these will be useful for breeding cold tolerance in rice.

Unlike traditional QTL mapping, GWAS enables the identification of rare alleles in natural populations and of high-resolution SNP markers (Gupta et al. 2014). Although Pan et al. (2015) and Fujino et al. (2015) recently reported the association mapping of cold tolerance QTLs in rice, the low resolution of SSR marker genotyping data affects the interval distance of the mapped QTLs. In this study, we mapped 67 QTLs associated with CTS in 295 rice accessions. By comparing the current and previously published results (Yang et al. 2015; Lv et al. 2016), we found that 56 of the 67 QTLs are newly identified and that only 11 are co-localized with genes of known function or with mapped QTLs (Fig. 2 and Additional file 3: Table S3). Within these mapped QTLs, we sequenced and genotyped the predicted candidate gene *Osryh1* on QTL *qCTS3-8*, and found a high correlation between the indel marker genotype and CTS phenotype, demonstrating that our mapping results are suitable for further characterization of CTS candidate genes.

Furthermore, we compared the QTL loci for CTS in both japonica and indica cultivars. The results showed that 34 loci in the indica group and 91 loci in the japonica group were mapped (data not shown), suggesting that more QTLs for CTS are accumulated in the japonica cultivars than that in the indica ones. This could be one of reasons why japonica cultivars are more tolerant to cold stress than other type of rice (Additional file 1: Table S1).

In the collection of RPD1 rice accessions, we found that 40 rice cultivars are highly tolerant to cold stress at the seedling stage (Additional file 1: Table S1 and Additional file 4: S4). In 40 selected highly tolerant cultivars, 87.5% (35/40) of cultivars are japonica rice including 20 TRJ and 15 TEJ types (Additional file 4: Table S4). Further allele frequency analysis with the 181 associated SNPs revealed that over 60% loci in the 40 cultivars are tolerant SNP types, and this is a 2-fold more than that in the 19 extremely sensitive cultivars (Fig. 2c and Additional file 4: Table S4), suggesting that cold tolerant alleles are highly enriched in the 40 cultivars.

Cold stress is one of the major limitations for rice production, especially in the double-season rice cropping regions. For example, in the early season in South China, farmers would like to grow rice earlier to gain more yield. However, cold weather in early spring usually forces the delay of first rice planting. Consequently this causes the delay of the second crop planting and thus affect flowering when cold weather starts in middle or late September. In our study, the identified cultivars with high cold tolerance will be useful germplasm for rice cold tolerance breeding, and some of the associated SNPs can be designed as molecular markers for cold tolerance breeding or used for fine mapping and cloning of cold tolerance QTLs.

## Conclusions

We evaluated the cold tolerance of 295 rice accessions in RDP1 collected from 82 countries at the seedling stage, and performed GWAS to map QTLs associated with CTS. The evaluations indicated that both temperate and tropical japonica rice cultivars are more tolerant to cold stress than IND and AUS rice. GWAS revealed that 67 QTLs are associated with CTS. Among them, 56 are novel loci. Our study has provided new information on the genetic structure of cold tolerance in rice, identified highly cold tolerant cultivars and CTS-associated SNP markers for rice improvement.

## Methods

### Plant Material and Plant Growth

About 30 seeds of each cultivar were germinated in Petri dishes in an incubator. The germinated seedlings were sown in pots containing soil and kept in a growth chamber. The standard growth conditions were 12 h of light at 25 °C with 75% humidity, and 12 h of dark at 20 °C with 70% humidity.

### Cold-stress Treatment and Evaluation of Cold Tolerance

Rice seedlings at 3rd leaf stage were subjected to cold-stress treatment at 8 °C for 3 days with 75% humidity and 12 h light/12 h dark. After seven days under standard growth conditions, the seedlings were scored for cold tolerance. The experiment was repeated three times under the same conditions.

The seedlings were scored for cold tolerance on a 1–9 scale as previously reported (Han and Zhang 2004; Li et al. 2006). For scores of 1 2, 3, 4, 5, 6, 7, 8, and 9, the percentage of dry and red leaves is ≤20, between 21 and 30, between 31 and 40, between 41 and 50, between 51 and 60, between 61 and 70, between 71 and 80, between 81 and 90, and > 90, respectively. The average of three replications was used for GWAS.

### Correlation Coefficient and Linear Regression Analysis

The cold tolerance scores of all the tested cultivars and their corresponding latitude values were used for the correlation analysis. The correlaton coefficient and linear regression programs in the Microsoft Excel software were used for the calculation.

### GWAS Mapping of Rice CTS QTLs

Tassel 3.0 software was used for GWAS of rice CTS QTLs. The public 44 K SNP genotype and cold-tolerance phenotype dataset were input into Tassel 3.0 software and assessed with the MLM (mixed linear model). The analysis procedure was same as that reported by Kang et al. (2016).

### Candidate Gene Prediction, Sequencing, and Sequence Alignment

The 500-kb reference sequence of the mapped CTS QTL regions was downloaded for gene annotation. Based on the annotation, the genes related to stresses were selected for selection of cold-stress candidate genes. Then, the corresponding candidate genes were cloned by PCR and sequenced in 20 cold-tolerant and 20 cold-sensitive rice cultivars. Sequence alignment was performed with the Clustal W program with the genes in the Nipponbare genome as a reference.

### Validation of Candidate Genes with Molecular Markers

The 27-bp indel difference between the cold-tolerant and cold-sensitive rice cultivars for the candidate gene LOC_Os03g0191400 was designed to be an indel maker using the conserved flanking sequences as primers (forward primer: AGAATGGTCCTGACAT CG; reversed primer: TTTGGTGGCTCCTCTTACGGG). The marker was further used to genotype 153 rice cultivars by PCR with the following PCR protocol: 40 cycles at 95 °C for 15 s, 50 °C for 30 s, and 72 °C for 20 s. PCR products were run on a 7% SDS-PAGE gel and stained with AgNO_3_.

## Additional files

Additional file 1: Table S1. The cold tolerance scores of the evaluated rice accessions. (DOCX 40 kb)
Additional file 2: Table S2. Cold tolerance distribution of the subpopulations in the rice diversity panel 1 (RDP1). (DOCX 44 kb)
Additional file 3: Table S3. QTL lists for rice cold tolerance at the seedling stage in the RDP1 accessions. (PDF 214 kb)
Additional file 4: Table S4. Allele frequency of the 181 significant SNPs in the 40 highly tolerant and the 19 extremely sensitive rice accessions. (DOCX 18 kb)
