# Supplementary material for: Genome-wide Association Mapping of Cold Tolerance Genes at the Seedling Stage in Rice
Source: Rice (N Y). 2016 Nov 15;9:61. doi: 10.1186/s12284-016-0133-2 (PMC5110459; doi:10.1186/s12284-016-0133-2)
Supplement: Additional file 1: Table S1. — The cold tolerance scores of the evaluated rice accessions. (DOCX 40 kb) [file 12284_2016_133_MOESM1_ESM.docx]

**Table S1.** The cold tolerance scores of the evaluated rice accessions.

| **NSFTV No.** | **Origin** | **Subgroup** | **CT Score** | **NSFTV No.** | **Origin** | **Subgroup** | **CT Score** |
| --- | --- | --- | --- | --- | --- | --- | --- |
| NSFTV_20a | Louisiana | ADMIX | 1 | NSFTV_171a | China | IND | 7 |
| NSFTV_619a | United States | ADMIX | 1 | NSFTV_203a | Malaysia | IND | 7 |
| NSFTV_55a | Iran | ADMIX | 2 | NSFTV_241a | Cuba | IND | 7 |
| NSFTV_114a | United States | ADMIX | 2 | NSFTV_269a | Kazakhstan | IND | 7 |
| NSFTV_128a | Australia | ADMIX | 2 | NSFTV_339a | Myanmar | IND | 7 |
| NSFTV_182a | United States | ADMIX | 2 | NSFTV_349a | China | IND | 7 |
| NSFTV_253a | Guinea | ADMIX | 2 | NSFTV_106a | China | IND | 8 |
| NSFTV_340a | Afghanistan | ADMIX | 2 | NSFTV_119a | Colombia | IND | 8 |
| NSFTV_343a | Argentina | ADMIX | 2 | NSFTV_129a | Taiwan | IND | 8 |
| NSFTV_387a | United States-CA | ADMIX | 2 | NSFTV_130a | Taiwan | IND | 8 |
| NSFTV_621a | United States | ADMIX | 2 | NSFTV_132a | Sri Lanka | IND | 8 |
| NSFTV_40a | Thailand | ADMIX | 3 | NSFTV_207a | Indonesia | IND | 8 |
| NSFTV_205a | Italy | ADMIX | 3 | NSFTV_209a | Gabon | IND | 8 |
| NSFTV_217a | Australia | ADMIX | 3 | NSFTV_313a | Bangladesh | IND | 8 |
| NSFTV_386a | United States | ADMIX | 3 | NSFTV_315a | Myanmar | IND | 8 |
| NSFTV_390a | United States | ADMIX | 3 | NSFTV_385a | United States | IND | 8 |
| NSFTV_352a | United States | ADMIX | 3 | NSFTV_71a | Philippines | IND | 9 |
| NSFTV_39a | Bangladesh | ADMIX | 4 | NSFTV_110a | India | IND | 9 |
| NSFTV_60a | Indonesia | ADMIX | 4 | NSFTV_172a | China | IND | 9 |
| NSFTV_166a | Madagascar | ADMIX | 4 | NSFTV_252a | Guinea | IND | 9 |
| NSFTV_218a | Australia | ADMIX | 4 | NSFTV_284a | Nepal | IND | 9 |
| NSFTV_237a | Colombia | ADMIX | 4 | NSFTV_298a | Sri Lanka | IND | 9 |
| NSFTV_266a | Japan | ADMIX | 4 | NSFTV_325a | Myanmar | IND | 9 |
| NSFTV_270a | Macedonia | ADMIX | 4 | NSFTV_124a | Philippines | IND | 9 |
| NSFTV_271a | Macedonia | ADMIX | 4 | NSFTV_612a | Colombia | IND | 9 |
| NSFTV_305a | Uruguay | ADMIX | 4 | NSFTV_83a | Japan | TEJ | 1 |
| NSFTV_335a | Myanmar | ADMIX | 4 | NSFTV_103a | Afghanistan | TEJ | 1 |
| NSFTV_344a | Bangladesh | ADMIX | 4 | NSFTV_115a | Pakistan | TEJ | 1 |
| NSFTV_364a | Japan | ADMIX | 4 | NSFTV_155a | China | TEJ | 1 |
| NSFTV_367a | Korea | ADMIX | 4 | NSFTV_204a | Italy | TEJ | 1 |
| NSFTV_87a | Indonesia | ADMIX | 5 | NSFTV_216a | Egypt | TEJ | 1 |
| NSFTV_140a | United States | ADMIX | 5 | NSFTV_245a | Egypt | TEJ | 1 |
| NSFTV_278a | Mexico | ADMIX | 5 | NSFTV_257a | Hungary | TEJ | 1 |
| NSFTV_100a | United States | ADMIX | 6 | NSFTV_281a | Morocco | TEJ | 1 |
| NSFTV_168a | Madagascar | ADMIX | 6 | NSFTV_282a | Morocco | TEJ | 1 |
| NSFTV_80a | Suriname | ADMIX | 7 | NSFTV_288a | Poland | TEJ | 1 |
| NSFTV_206a | Madagascar | ADMIX | 7 | NSFTV_300a | Suriname | TEJ | 1 |
| NSFTV_210a | Japan | ADMIX | 7 | NSFTV_306a | Uzbekistan | TEJ | 1 |
| NSFTV_227a | Burkina Faso | ADMIX | 7 | NSFTV_334a | Thailand | TEJ | 1 |
| NSFTV_293a | Senegal | ADMIX | 7 | NSFTV_380a | Taiwan | TEJ | 1 |
| NSFTV_358a | India | ADMIX | 7 | NSFTV_1a | Italy | TEJ | 2 |
| NSFTV_643a | China | ADMIX | 7 | NSFTV_9a | India | TEJ | 2 |
| NSFTV_259a | Iran | ADMIX | 8 | NSFTV_133a | Japan | TEJ | 2 |
| NSFTV_249a | Former Yugoslavia | ADMIX | 9 | NSFTV_143a | Japan | TEJ | 2 |
| NSFTV_626a | United States-CA | ADMIX | 9 | NSFTV_154a | China | TEJ | 2 |
| NSFTV_634a | Iran | AROMATIC | 1 | NSFTV_157a | Taiwan | TEJ | 2 |
| NSFTV_12a | Pakistan | AROMATIC | 3 | NSFTV_158a | Taiwan | TEJ | 2 |
| NSFTV_5a | India | AROMATIC | 4 | NSFTV_179a | France | TEJ | 2 |
| NSFTV_45a | Iran | AROMATIC | 4 | NSFTV_184a | Spain | TEJ | 2 |
| NSFTV_112a | India | AROMATIC | 4 | NSFTV_224a | Bulgaria | TEJ | 2 |
| NSFTV_160a | Iran | AROMATIC | 4 | NSFTV_247a | Former Soviet Union | TEJ | 2 |
| NSFTV_373a | Peru | AROMATIC | 4 | NSFTV_248a | Former Soviet Union | TEJ | 2 |
| NSFTV_53a | Iran | AROMATIC | 5 | NSFTV_263a | Italy | TEJ | 2 |
| NSFTV_260a | Iran | AROMATIC | 5 | NSFTV_267a | Japan | TEJ | 2 |
| NSFTV_16a | Brazil | AROMATIC | 7 | NSFTV_283a | Mozambique | TEJ | 2 |
| NSFTV_93a | Madagascar | AROMATIC | 7 | NSFTV_291a | Romania | TEJ | 2 |
| NSFTV_105a | Iran | AUS | 3 | NSFTV_307a | Uzbekistan | TEJ | 2 |
| NSFTV_354a | India | AUS | 3 | NSFTV_32a | South Korea | TEJ | 3 |
| NSFTV_18a | India | AUS | 4 | NSFTV_52a | Taiwan | TEJ | 3 |
| NSFTV_33a | Taiwan | AUS | 4 | NSFTV_56a | South Korea | TEJ | 3 |
| NSFTV_261a | Iraq | AUS | 4 | NSFTV_104a | Japan | TEJ | 3 |
| NSFTV_322a | Bangladesh | AUS | 4 | NSFTV_173a | Japan | TEJ | 3 |
| NSFTV_631a | India | AUS | 4 | NSFTV_180a | Peru | TEJ | 3 |
| NSFTV_19a | India | AUS | 5 | NSFTV_186a | South Korea | TEJ | 3 |
| NSFTV_246a | Fiji | AUS | 5 | NSFTV_243a | Ecuador | TEJ | 3 |
| NSFTV_312a | Bangladesh | AUS | 5 | NSFTV_250a | France | TEJ | 3 |
| NSFTV_359a | India | AUS | 5 | NSFTV_265a | Italy | TEJ | 3 |
| NSFTV_372a | Pakistan | AUS | 5 | NSFTV_275a | Malaysia | TEJ | 3 |
| NSFTV_378a | Sri Lanka | AUS | 5 | NSFTV_289a | Portugal | TEJ | 3 |
| NSFTV_13a | Pakistan | AUS | 6 | NSFTV_290a | Puerto Rico | TEJ | 3 |
| NSFTV_49a | Bangladesh | AUS | 6 | NSFTV_295a | Spain | TEJ | 3 |
| NSFTV_81a | India | AUS | 6 | NSFTV_296a | Spain | TEJ | 3 |
| NSFTV_88a | Thailand | AUS | 6 | NSFTV_303a | Tanzania | TEJ | 3 |
| NSFTV_228a | Chad | AUS | 6 | NSFTV_311a | Thailand | TEJ | 3 |
| NSFTV_262a | Iraq | AUS | 6 | NSFTV_333a | Thailand | TEJ | 3 |
| NSFTV_341a | Afghanistan | AUS | 6 | NSFTV_363a | Japan | TEJ | 3 |
| NSFTV_371a | Pakistan | AUS | 6 | NSFTV_67a | China | TEJ | 4 |
| NSFTV_642a | India | AUS | 6 | NSFTV_292a | Romania | TEJ | 4 |
| NSFTV_4a | India | AUS | 7 | NSFTV_297a | Spain | TEJ | 4 |
| NSFTV_44a | Bangladesh | AUS | 7 | NSFTV_616a | Taiwan | TEJ | 4 |
| NSFTV_58a | Afghanistan | AUS | 7 | NSFTV_118a | Chile | TEJ | 6 |
| NSFTV_78a | India | AUS | 7 | NSFTV_86a | Thailand | TEJ | 7 |
| NSFTV_131a | Bhutan | AUS | 7 | NSFTV_633a | Japan | TEJ | 9 |
| NSFTV_200a | Pakistan | AUS | 7 | NSFTV_8a | Philippines | TRJ | 1 |
| NSFTV_276a | Mali | AUS | 7 | NSFTV_14a | India | TRJ | 1 |
| NSFTV_316a | Bangladesh | AUS | 7 | NSFTV_22a | Taiwan | TRJ | 1 |
| NSFTV_317a | Bangladesh | AUS | 7 | NSFTV_27a | Pakistan | TRJ | 1 |
| NSFTV_320a | Bangladesh | AUS | 7 | NSFTV_92a | Philippines | TRJ | 1 |
| NSFTV_321a | Bangladesh | AUS | 7 | NSFTV_98a | United States_CA | TRJ | 1 |
| NSFTV_324a | Bangladesh | AUS | 7 | NSFTV_99a | Liberia | TRJ | 1 |
| NSFTV_328a | Bangladesh | AUS | 7 | NSFTV_107a | Bangladesh | TRJ | 1 |
| NSFTV_336a | Myanmar | AUS | 7 | NSFTV_108a | Guinea | TRJ | 1 |
| NSFTV_346a | Bangladesh | AUS | 7 | NSFTV_116a | Pakistan | TRJ | 1 |
| NSFTV_357a | India | AUS | 7 | NSFTV_174a | Philippines | TRJ | 1 |
| NSFTV_360a | India | AUS | 7 | NSFTV_195a | Cote D'Ivoire | TRJ | 1 |
| NSFTV_50a | Bangladesh | AUS | 8 | NSFTV_223a | Brazil | TRJ | 1 |
| NSFTV_153a | India | AUS | 8 | NSFTV_226a | Burkina Faso | TRJ | 1 |
| NSFTV_314a | Bangladesh | AUS | 8 | NSFTV_239a | Cote D'Ivoire | TRJ | 1 |
| NSFTV_319a | Bangladesh | AUS | 8 | NSFTV_240a | Cote D'Ivoire | TRJ | 1 |
| NSFTV_326a | Bangladesh | AUS | 8 | NSFTV_251a | Argentina | TRJ | 1 |
| NSFTV_327a | Bangladesh | AUS | 8 | NSFTV_286a | Nigeria | TRJ | 1 |
| NSFTV_329a | Bangladesh | AUS | 8 | NSFTV_396a | United States | TRJ | 1 |
| NSFTV_331a | Thailand | AUS | 8 | NSFTV_397a | United States | TRJ | 1 |
| NSFTV_345a | Bangladesh | AUS | 8 | NSFTV_24a | United States | TRJ | 2 |
| NSFTV_85a | India | AUS | 9 | NSFTV_25a | United States | TRJ | 2 |
| NSFTV_152a | India | AUS | 9 | NSFTV_75a | Indonesia | TRJ | 2 |
| NSFTV_330a | Thailand | AUS | 9 | NSFTV_84a | Indonesia | TRJ | 2 |
| NSFTV_6a 5 | India | AUS | 5 | NSFTV_89a | Thailand | TRJ | 2 |
| NSFTV_17a | Philippines | IND | 1 | NSFTV_101a | United States | TRJ | 2 |
| NSFTV_161a | China | IND | 1 | NSFTV_139a | United States | TRJ | 2 |
| NSFTV_76a | India | IND | 2 | NSFTV_149a | Philippines | TRJ | 2 |
| NSFTV_156a | Taiwan | IND | 2 | NSFTV_150a | Egypt | TRJ | 2 |
| NSFTV_375a | China | IND | 2 | NSFTV_199a | Bolivia | TRJ | 2 |
| NSFTV_628a | China | IND | 2 | NSFTV_258a | Indonesia | TRJ | 2 |
| NSFTV_630a | Korea | IND | 2 | NSFTV_285a | Nigeria | TRJ | 2 |
| NSFTV_625a | Philippines | IND | 2 | NSFTV_377a | Puerto Rico | TRJ | 2 |
| NSFTV_3a | China | IND | 3 | NSFTV_384a | TURKEY | TRJ | 2 |
| NSFTV_72a | Philippines | IND | 3 | NSFTV_391a | United States | TRJ | 2 |
| NSFTV_21a | Australia | IND | 4 | NSFTV_392a | United States | TRJ | 2 |
| NSFTV_208a | India | IND | 4 | NSFTV_629a | Philipppines | TRJ | 2 |
| NSFTV_622a | China | IND | 4 | NSFTV_26a | United States | TRJ | 3 |
| NSFTV_641a | China | IND | 4 | NSFTV_37a | Cuba | TRJ | 3 |
| NSFTV_43a | Taiwan | IND | 5 | NSFTV_46a | Brazil | TRJ | 3 |
| NSFTV_66a | Taiwan | IND | 5 | NSFTV_54a | United States | TRJ | 3 |
| NSFTV_162a | India | IND | 5 | NSFTV_59a | Indonesia | TRJ | 3 |
| NSFTV_196a | Mali | IND | 5 | NSFTV_65a | Honduras | TRJ | 3 |
| NSFTV_222a | Brazil | IND | 5 | NSFTV_70a | Haiti | TRJ | 3 |
| NSFTV_254a | Hong Kong | IND | 5 | NSFTV_120a | Nigeria | TRJ | 3 |
| NSFTV_617a | China | IND | 5 | NSFTV_135a | Zaire | TRJ | 3 |
| NSFTV_57a | Iran | IND | 6 | NSFTV_165a | Indonesia | TRJ | 3 |
| NSFTV_61a | China | IND | 6 | NSFTV_185a | Belize | TRJ | 3 |
| NSFTV_90a | Taiwan | IND | 6 | NSFTV_187a | United States | TRJ | 3 |
| NSFTV_97a | China | IND | 6 | NSFTV_188a | Bulgaria | TRJ | 3 |
| NSFTV_137a | Vietnam | IND | 6 | NSFTV_198a | Bulgaria | TRJ | 3 |
| NSFTV_141a | Indonesia | IND | 6 | NSFTV_213a | Jamaica | TRJ | 3 |
| NSFTV_189a | Venezuela | IND | 6 | NSFTV_242a | Dominican Republic | TRJ | 3 |
| NSFTV_234a | China | IND | 6 | NSFTV_310a | Zaire | TRJ | 3 |
| NSFTV_235a | China | IND | 6 | NSFTV_347a | Belize | TRJ | 3 |
| NSFTV_255a | Hong Kong | IND | 6 | NSFTV_381a | Taiwan | TRJ | 3 |
| NSFTV_299a | Suriname | IND | 6 | NSFTV_395a | Zaire | TRJ | 3 |
| NSFTV_337a | Bangladesh | IND | 6 | NSFTV_69a | Brazil | TRJ | 4 |
| NSFTV_348a | China | IND | 6 | NSFTV_618a | United States | TRJ | 5 |
| NSFTV_356a | India | IND | 6 | NSFTV_620a | United States | TRJ | 6 |
| NSFTV_74a | Brazil | IND | 7 | NSFTV_308a | Venezuela | TRJ | 7 |
| NSFTV_109a | India | IND | 7 | NSFTV_350a | Colombia | TRJ | 7 |
| NSFTV_117a | Taiwan | IND | 7 | NSFTV_353a | Guatemala | TRJ | 8 |
| NSFTV_123a | Taiwan | IND | 7 | NSFTV_623a | United States | TRJ | 9 |
| NSFTV_125a | China | IND | 7 |  |  |  |  |
