# Supplementary material for: Genome-wide Association Mapping of Cold Tolerance Genes at the Seedling Stage in Rice
Source: Rice (N Y). 2016 Nov 15;9:61. doi: 10.1186/s12284-016-0133-2 (PMC5110459; doi:10.1186/s12284-016-0133-2)
Supplement: Additional file 2: Table S2. — Cold tolerance distribution of the subpopulations in the rice diversity panel 1 (RDP1). (DOCX 44 kb) [file 12284_2016_133_MOESM2_ESM.docx]

**Table S2.** Cold tolerance distribution of the subpopulations in the rice diversity panel 1 (RDP1).

| **Score** | **TRJ** | **TEJ** | **ADMIX** | **AROMATIC** | **IND** | **AUS** | **Total** |
| --- | --- | --- | --- | --- | --- | --- | --- |
| **1** | 20 | 15 | 2 | 1 | 2 | 0 | 40 |
| **2** | 17 | 17 | 9 | 0 | 6 | 0 | 49 |
| **3** | 20 | 19 | 6 | 1 | 2 | 2 | 50 |
| **4** | 1 | 4 | 13 | 5 | 4 | 5 | 32 |
| **5** | 1 | 0 | 3 | 2 | 7 | 7 | 20 |
| **6** | 1 | 1 | 2 | 0 | 14 | 9 | 27 |
| **7** | 2 | 1 | 7 | 2 | 11 | 17 | 40 |
| **8** | 1 | 0 | 1 | 0 | 10 | 9 | 21 |
| **9** | 1 | 1 | 2 | 0 | 9 | 3 | 16 |
| **Total** | 64 | 58 | 45 | 11 | 65 | 52 | 295 |
