# Supplementary material for: Genome-wide Association Mapping of Cold Tolerance Genes at the Seedling Stage in Rice
Source: Rice (N Y). 2016 Nov 15;9:61. doi: 10.1186/s12284-016-0133-2 (PMC5110459; doi:10.1186/s12284-016-0133-2)
Supplement: Additional file 3: Table S3. — QTL lists for rice cold tolerance at the seedling stage in the RDP1 accessions. (PDF 214 kb) [file 12284_2016_133_MOESM3_ESM.pdf]

Table S3. QTL lists for rice cold tolerance at seedling stage in RDP1 accessions.

Chromosome	SNP Site	Allele	Tolerant allelic SNP	Minor Allele Frequency （%）	-Log(p-value)	QTL	Colocalized QTLs or genes
1	7392085	C/T	CC	24.3	3.13	*qCTS1-1*	
1	19180885	C/T	TT	1.7	3.99	*qCTS1-2*	
1	20261058	A/G	AA	10.6	3.27	*qCTS1-3*	
1	41385263	C/T	TT	36.2	3.28	*qCTS1-4*	
1	41676236	A/G	AA	44.4	3.13	*qCTS1-5*	
1	41840550	C/T	TT	29.2	3.28		
1	41859177	A/T	AA	21.6	3.88		
1	42253613	A/G	AA	8.8	3.11	*qCTS1-6*	OsDREB1F LOC_Os01g73770 Chr01:42727426-42728363
1	42438945	A/C	CC	8.5	3.23		
2	74241	A/G	GG	3.6	4.05	*qCTS2-1*	
2	4797319	A/G	AA	38.8	3.12	*qCTS2-2*	
2	15096404	A/G	AA	4.8	3.54	*qCTS2-3*	
2	33750096	C/T	CC	40.8	3.06	*qCTS2-4*	
2	35836085	C/T	TT	11.0	3.24		
2	35929494	A/T	TT	16.8	3.84		
3	563320	A/G	GG	44.9	3.32	*qCTS3-1*	qLTG3-1, LOC_Os03g01320 Chr03:219977-221070
3	576440	A/T	TT	44.1	3.25		
3	604512	C/T	CC	44.8	3.54		
3	958590	A/G	GG	43.9	3.04	*qCTS3-2*	
3	995211	G/T	TT	43.9	3.52		
3	1070899	A/C	CC	43.6	3.53		

3	1075138	A/T	TT	44.0	3.36		
3	1106895	A/G	GG	44.5	3.46		
3	1207632	C/T	TT	31.2	3.38	*qCTS3-3*	
3	1243161	A/T	AA	5.2	3.99		
3	2110407	A/T	TT	41.2	3.81	*qCTS3-4*	
3	2129834	C/T	TT	41.2	3.79		
3	2680925	C/T	CC	33.0	4.88	*qCTS3-5*	
3	2774292	A/G	AA	44.5	3.16		
3	2810628	C/T	TT	44.7	3.16		
3	2869149	A/G	GG	44.6	3.18		
3	2880288	C/T	CC	37.2	3.05		
3	2907220	A/G	GG	44.1	3.34	*qCTS3-6*	
3	2918213	C/T	TT	44.5	3.16		
3	2962574	C/G	GG	44.4	3.19		
3	2984802	C/G	CC	44.5	3.16		
3	3003744	C/G	GG	44.3	3.31		
3	3032938	C/T	TT	43.8	3.44		
3	3211019	C/T	CC	36.8	3.91	*qCTS3-7*	
3	3214132	A/G	GG	43.2	4.24		
3	3273074	A/C	AA	44.5	3.16		
3	3484068	C/T	TT	3.2	3.35	*qCTS3-8*	
3	3508770	A/G	AA	45.8	3.12		
3	3530718	A/T	TT	45.4	3.17		
3	3557931	A/G	AA	45.5	3.17		
3	3595014	A/T	AA	45.9	3.17		

3	3650136	G/T	TT	45.9	4.44		
3	3668304	A/C	CC	45.8	4.68		
3	3761773	A/G	AA	45.6	3.05	*qCTS3-9*	candidate gene *Osryh1* locus
3	3763778	G/T	GG	45.5	4.43		
3	3776771	C/T	CC	43.5	4.02		
3	8192574	A/T	AA	4.1	3.26	*qCTS3-10*	
3	8434823	C/T	TT	5.0	3.15		
3	22540833	C/T	TT	15.9	3.14	*qCTS3-11*	
3	27608937	A/G	AA	37.0	3.69	*qCTS3-12*	
3	27624561	C/G	TT	25.1	3.01		
3	27630098	C/T	CC	15.0	3.00		
3	27636236	A/G	GG	26.4	3.17		
3	27653586	G/T	TT	13.6	3.09		
3	27658570	C/T	TT	33.2	3.16		
3	31924530	A/G	AA	6.9	3.19	*qCTS3-13*	
4	5850082	A/T	TT	25.3	3.65	*qCTS4-1*	
5	1970193	A/G	GG	43.9	3.03	*qCTS5-1*	Locus 52 1864564-2057572 (Lv et al., 2016); qSV-5 (Zhang et al., 2005)
5	1972789	A/G	AA	37.1	3.29		
5	6171822	A/C	CC	8.9	3.58	*qCTS5-2*	
5	8040996	A/C	AA	33.7	3.29	*qCTS5-3*	
5	24574651	C/T	CC	42.6	3.29	*qCTS5-4*	
5	24616513	A/G	GG	42.9	3.33		
5	24633331	A/G	AA	41.2	3.17		
5	24662609	C/T	TT	43.1	3.04		
5	24662707	A/G	AA	42.6	3.06		

5	24664730	C/T	TT	43.0	3.32		
5	24702049	A/G	AA	43.1	3.04		
5	24729412	A/G	GG	43.1	3.09		
5	24735766	C/G	GG	42.9	3.04		
5	24772917	C/G	GG	43.1	3.04		
5	24899960	G/T	GG	43.1	3.04	*qCTS5-5*	
5	24900315	C/T	CC	43.1	3.04		
5	24902358	A/G	AA	43.1	3.04		
5	24932779	C/T	TT	42.5	3.12		
5	24955108	A/G	AA	42.9	3.04		
5	25036987	C/T	CC	42.3	3.90		
5	25037918	A/C	CC	42.6	3.86		
5	25039078	C/T	TT	42.4	3.87		
5	25042012	C/T	TT	25.9	3.97		
5	25042504	C/T	CC	41.3	4.64		
5	25098060	C/T	TT	41.8	4.56		
5	25099569	A/G	GG	42.1	4.62		
5	25114753	A/G	GG	42.1	4.62	*qCTS5-6*	
5	25152991	C/T	CC	42.1	4.62		
5	25154581	A/C	AA	42.0	4.82		
5	25159948	A/T	AA	42.2	4.61		
5	25215645	C/T	TT	41.9	4.72		
5	25240733	A/G	GG	42.1	4.74		
5	25661962	A/C	AA	41.2	3.04	*qCTS5-7*	
5	25663189	A/G	GG	41.6	3.07		

5	25720662	A/G	AA	41.4	3.10		
5	25726104	A/G	GG	41.6	3.07		
5	25753030	A/T	AA	41.5	3.08		
5	25888055	A/G	GG	41.4	3.07	*qCTS5-8*	RM3321-RM1054, 25.78-29.16 (Yang et al., 2015)
5	25888180	A/G	AA	41.6	3.07		
5	25888522	G/T	GG	41.6	3.07		
5	25903798	A/G	TT	41.2	3.07		
5	25964590	A/G	AA	41.6	3.07		
5	25990922	G/T	CC	41.6	3.07		
5	26038047	A/T	AA	41.6	3.07		
5	26073607	C/T	TT	41.9	5.03		
5	26085402	A/G	TT	29.4	3.19		
5	26092015	G/T	TT	41.2	5.05		
5	26099117	A/G	AA	46.6	4.25		
5	29432186	C/T	TT	4.5	3.84	*qCTS5-9*	
5	29471226	C/T	GG	4.8	3.63		
6	4733861	C/T	TT	13.1	3.39	*qCTS6-1*	
6	6228690	A/G	AA	25.6	3.18	*qCTS6-2*	
6	10452388	C/T	CC	25.5	3.46	*qCTS6-3*	
6	10964887	A/G	GG	10.0	3.60	*qCTS6-4*	
6	23767614	G/T	GG	36.8	3.22	*qCTS6-5*	OsSPX1 LOC_Os06g40120 Chr06:23875408-23879965
6	23808753	C/T	CC	31.7	3.07		
6	23809683	C/T	TT	31.9	3.19		
6	23845956	A/T	AA	31.7	3.03		
6	23876111	C/T	TT	30.6	3.52		

6	23877977	C/T	TT	77.1	3.18		
7	914685	C/T	CC	41.4	3.30	*qCTS7-1*	
7	17875297	A/G	GG	24.1	3.09	*qCTS7-2*	
7	18202939	A/C	CC	33.7	4.67	*qCTS7-3*	
7	23783174	C/T	CC	15.4	3.12	*qCTS7-4*	
7	29049032	C/T	CC	21.8	3.34	*qCTS7-5*	RM234-RM22187, 25.47-29.60 (Yang et al., 2015) ; OsFAD8 LOC_Os07g49310 Chr07:29531227-29534135
7	29151249	G/T	GG	25.2	3.83		
7	29164601	C/T	TT	22.1	4.10		
7	29220680	A/G	AA	22.1	4.08		
8	8883364	A/G	AA	3.5	3.44	*qCTS8-1*	
8	10142772	C/T	CC	3.1	4.21	*qCTS82*	
8	10160968	A/G	GG	4.8	3.33		
8	10161067	A/T	AA	37.3	3.19		
8	10164021	G/T	TT	37.0	3.18		
8	10213987	C/T	TT	36.5	3.33		
8	10506862	A/G	GG	4.5	3.16	*qCTS8-3*	
8	20385371	A/G	AA	3.9	3.82	*qCTS8-4*	
9	1141448	C/T	CC	27.9	3.49	*qCTS9-1*	OsCYL4a LOC_Os09g02270 Chr09:920565-926016
9	1257769	A/G	AA	28.6	3.04		
9	1260312	A/G	GG	29.5	3.23		
9	1706118	C/T	CC	28.0	3.25	*qCTS9-2*	
9	2176232	A/T	TT	27.3	3.04	*qCTS9-3*	
9	2227843	C/T	TT	27.5	3.16		
9	3375476	C/T	CC	31.4	3.29		
9	3869544	A/G	GG	31.6	3.11	*qCTS9-4*	

9	3869635	G/T	TT	30.2	3.28		
9	4283153	C/G	CC	30.3	3.12	*qCTS9-5*	Locus92 RLC 4304471-4488259 (Lv et al., 2016); Clr9 (Oh et al., 2004)
9	4289803	A/G	GG	28.9	3.56		
9	4349760	A/C	AA	31.0	3.24		
9	4767843	C/G	TT	31.4	3.04	*qCTS9-6*	
9	6403821	A/T	AA	13.6	3.06	*qCTS9-7*	
9	16181745	C/T	CC	3.8	3.26	*qCTS9-8*	
9	17697057	C/T	GG	43.6	3.15	*qCTS9-10*	
10	5690202	C/T	CC	9.9	3.59	*qCTS10-1*	
11	5902436	A/G	AA	23.7	3.25	*qCTS11-1*	RM5599-RM3701, 3.82-8.10 (Yang et al., 2015)
11	6478793	A/G	GG	25.5	3.60	*qCTS11-2*	
11	7249274	A/G	GG	36.2	3.64	*qCTS11-3*	
11	9163345	A/G	GG	17.9	3.45	*qCTS11-4*	
11	17519565	A/G	AA	28.6	3.27	*qCTS11-5*	
11	19897968	A/G	AA	31.1	3.49	*qCTS11-6*	RM229-RM21, 18.41-21.47 (Yang et al., 2015) ; Locus 112 19810529-20004427;qLTG-11-1/qSCT11 Jiang et et al, 2006; Kim et al., 2014
11	21756289	C/T	TT	33.2	3.39	*qCTS11-7*	RM21-RM206, 21.47-22.01 (Yang et al., 2015)
11	21779484	A/G	AA	39.3	3.87		
11	21953047	G/T	TT	2.6	3.08		
11	21995775	G/T	TT	0.7	3.39	*qCTS11-8*	
11	22103236	C/T	CC	1.2	3.30		
11	22114374	A/G	GG	3.4	3.10		
11	22115297	G/T	GG	16.8	3.21		

11	22184307	A/G	AA	0.7	3.76		
11	22186102	A/C	CC	1.0	3.75		
11	22186231	A/T	AA	1.0	3.71		
11	22188699	C/T	TT	1.0	3.70		
11	22189254	A/G	AA	1.0	3.74		
11	22189449	C/T	CC	1.0	3.75		
11	22189552	A/G	AA	1.0	3.75		
11	22190448	C/T	CC	1.0	3.75		
11	24958836	C/T	CC	39.9	3.67	*qCTS11-9*	
11	25426057	C/T	TT	26.2	3.36	*qCTS11-10*	
11	25432012	C/T	CC	36.3	4.52		
11	25490130	C/G	GG	25.3	4.53		
11	25560824	C/T	TT	33.0	4.24		
