# Supplementary material for: Genome-wide Association Mapping of Cold Tolerance Genes at the Seedling Stage in Rice
Source: Rice (N Y). 2016 Nov 15;9:61. doi: 10.1186/s12284-016-0133-2 (PMC5110459; doi:10.1186/s12284-016-0133-2)
Supplement: Additional file 4: Table S4. — Allele frequency of the 181 significant SNPs in the 40 highly tolerant and the 19 extremely sensitive rice accessions. (DOCX 18 kb) [file 12284_2016_133_MOESM4_ESM.docx]

**Table S4.** Allele frequency of 181 significant SNPs in 40 highly tolerant and 19 extreamly senstive rice accessions.

| **Accession No.** | **Origin** | **Subspecies** | **Cold tolerance scores** | **Percentage of T genotype (%)** | **Percentage of S genotype (%)** | **Percentage of NA genotype (%)** |
| --- | --- | --- | --- | --- | --- | --- |
| NSFTV_8a | Philippines | TRJ | 1 | 68.0 | 27.6 | 4.4 |
| NSFTV_14a | India | TRJ | 1 | 69.1 | 28.7 | 2.2 |
| NSFTV_17a | Philippines | IND | 1 | 44.2 | 53.0 | 2.8 |
| NSFTV_20a | Louisiana | ADMIX | 1 | 69.1 | 30.9 | 0.0 |
| NSFTV_22a | Taiwan | TRJ | 1 | 68.5 | 27.1 | 4.4 |
| NSFTV_27a | Pakistan | TRJ | 1 | 70.2 | 29.3 | 0.6 |
| NSFTV_83a | Japan | TEJ | 1 | 60.8 | 39.2 | 0.0 |
| NSFTV_92a | Philippines | TRJ | 1 | 68.5 | 28.2 | 3.3 |
| NSFTV_98a | United States_CA | TRJ | 1 | 66.9 | 31.5 | 1.7 |
| NSFTV_99a | Liberia | TRJ | 1 | 71.3 | 28.2 | 0.6 |
| NSFTV_103a | Afghanistan | TEJ | 1 | 60.2 | 39.2 | 0.6 |
| NSFTV_107a | Bangladesh | TRJ | 1 | 70.2 | 28.7 | 1.1 |
| NSFTV_108a | Guinea | TRJ | 1 | 69.6 | 29.3 | 1.1 |
| NSFTV_115a | Pakistan | TEJ | 1 | 61.3 | 34.3 | 4.4 |
| NSFTV_116a | Pakistan | TRJ | 1 | 69.6 | 28.7 | 1.7 |
| NSFTV_155a | China | TEJ | 1 | 65.2 | 34.3 | 0.6 |
| NSFTV_161a | China | IND | 1 | 56.9 | 39.2 | 3.9 |
| NSFTV_174a | Philippines | TRJ | 1 | 68.5 | 30.4 | 1.1 |
| NSFTV_195a | Cote D'Ivoire | TRJ | 1 | 70.7 | 27.6 | 1.7 |
| NSFTV_204a | Italy | TEJ | 1 | 65.2 | 34.8 | 0.0 |
| NSFTV_223a | Brazil | TRJ | 1 | 70.7 | 28.2 | 1.1 |
| NSFTV_226a | Burkina Faso | TRJ | 1 | 58.0 | 27.6 | 14.4 |
| NSFTV_240a | Cote D'Ivoire | TRJ | 1 | 67.4 | 26.5 | 6.1 |
| NSFTV_251a | Argentina | TRJ | 1 | 70.7 | 27.1 | 2.2 |
| NSFTV_257a | Hungary | TEJ | 1 | 60.8 | 33.7 | 5.5 |
| NSFTV_282a | Morocco | TEJ | 1 | 64.6 | 34.8 | 0.6 |
| NSFTV_286a | Nigeria | TRJ | 1 | 64.6 | 26.5 | 8.8 |
| NSFTV_288a | Poland | TEJ | 1 | 64.1 | 34.3 | 1.7 |
| NSFTV_300a | Suriname | TEJ | 1 | 66.9 | 32.0 | 1.1 |
| NSFTV_306a | Uzbekistan | TEJ | 1 | 63.5 | 34.8 | 1.7 |
| NSFTV_334a | Thailand | TEJ | 1 | 56.4 | 35.9 | 7.7 |
| NSFTV_380a | Taiwan | TEJ | 1 | 59.1 | 39.8 | 1.1 |
| NSFTV_396a | United States | TRJ | 1 | 70.2 | 28.2 | 1.7 |
| NSFTV_619a | United States | ADMIX | 1 | 66.9 | 28.7 | 4.4 |
| NSFTV_245a | Egypt | TEJ | 1 | 64.6 | 32.6 | 2.8 |
| NSFTV_281a | Morocco | TEJ | 1 | 60.8 | 38.7 | 0.6 |
| NSFTV_634a | Iran | AROMATIC | 1 | 25.4 | 68.0 | 6.6 |
| NSFTV_239a | Cote D'Ivoire | TRJ | 1 | 71.8 | 28.2 | 0.0 |
| NSFTV_397a | United States | TRJ | 1 | 70.7 | 28.7 | 0.6 |
| NSFTV_216a | Egypt | TEJ | 1 | 59.7 | 39.2 | 1.1 |
| NSFTV_71a | Philippines | IND | 9 | 26.0 | 69.1 | 5.0 |
| NSFTV_85a | India | AUS | 9 | 30.9 | 65.7 | 3.3 |
| NSFTV_110a | India | IND | 9 | 28.7 | 66.3 | 5.0 |
| NSFTV_124a | Philippines | IND | 9 | 34.8 | 58.6 | 6.6 |
| NSFTV_152a | India | AUS | 9 | 29.3 | 67.4 | 3.3 |
| NSFTV_252a | Guinea | IND | 9 | 24.9 | 59.7 | 15.5 |
| NSFTV_298a | Sri Lanka | IND | 9 | 23.2 | 71.8 | 5.0 |
| NSFTV_325a | Myanmar | IND | 9 | 29.3 | 66.9 | 3.9 |
| NSFTV_330a | Thailand | AUS | 9 | 30.4 | 65.7 | 3.9 |
| NSFTV_172a | China | IND | 9 | 26.5 | 71.3 | 2.2 |
| NSFTV_284a | Nepal | IND | 9 | 25.4 | 69.1 | 5.5 |
| NSFTV_623a | United States | TRJ | 9 | 25.4 | 69.6 | 5.0 |
| NSFTV_626a | United States-CA | ADMIX | 9 | 21.5 | 71.3 | 7.2 |
| NSFTV_633a | Japan | TEJ | 9 | 21.5 | 64.1 | 14.4 |
| NSFTV_249a | Former Yugoslavia | ADMIX | 9 | 24.9 | 58.6 | 16.6 |

**Note:** T is tolerant, S represents sensitive, NA means unknown genotype. TRJ means
